# Impact of indoor Air Pollution on the Linear growth of children in Jimma, Ethiopia

**DOI:** 10.1186/s12889-024-17975-3

**Published:** 2024-02-16

**Authors:** Elias Mulat, Dessalegn Tamiru, Kalkidan Hassen Abate

**Affiliations:** 1 Department of Biomedical Sciences, Institute of Health, Jimma University, Jimma, Ethiopia; 2 Department of Nutrition and Dietetics, Food and Nutrition Research Institute, Jimma University, Jimma, Ethiopia

**Keywords:** Indoor air pollution, Linear growth, Children, Ethiopia

## Abstract

**Background:**

Stunting in children is the term for reduced linear growth and development, which is frequently brought on by a persistently inadequate diet, recurrent infections and chronic diseases or poor health conditions. Apart from the classic covariates of stunting, which include diet and illness, the relative contribution of household air pollution to chronic nutrition conditions is least studied. Hence, this study is conducted to investigate the impact of household air pollution on the linear growth of under-five children in Jimma town, Ethiopia.

**Methods:**

A prospective cohort study was employed to collect data from 280 under-five children who lived in households using solid fuel (exposed group, *n* = 140) and clean fuel (unexposed group, *n* = 140). Height-for-age Z scores were compared in both groups over a 12-month follow-up period. The difference in differences estimators were used for comparison of changes in the height-for-age Z scores from baseline to end line in exposed and non-exposed groups. The independent effect of the use of solid fuels on height-for-age Z scores was analyzed through a multivariable linear regression model. Statistical Significances were declared at *P* < 0.05 and 95% CI level.

**Results:**

In an unadjusted model (Model 1), compared with the clean fuel type, the mean difference in the height-for-age Z score of children in households using solid fuel was lower by 0.54 (-0.54, 95% CI -0.97, -0.12, *P* = 0.011). The beta coefficient remained negative after adjusting for age and sex (Model 2 -0.543, 95% CI -1.373, -0.563) and sociodemographic variables (Model 3: -0.543, 95% CI -1.362, -0.575). In the final model (Model 4), which adjusted for wealth quantile, dietary practice, water, sanitation and hygiene status and household food insecurity access scale, the beta coefficient held the same and significant (beta: -0.543, 95% CI -1.357, -0.579, *P* < 0.001). Higher HAZ scores were observed among female child (β: = 0.48, 95%CI: 0.28, 0.69), Child with father attended higher education (β: = 0.304 95%CI: 0.304, 95% CI 0.19, 0.41) as compared to male gender and those who did not attend a formal education, respectively. In contrast, child living in households with poor hygiene practices had lower HAZ score (β: -0.226, 95% CI: -0.449, -0.003), *P* < 0.001.

**Conclusions:**

Exposure to indoor air pollution was inversely related to linear growth. Furthermore, sex, educational status and hygiene were found relevant predictors of linear growth. In such a setting, there is a need to step up efforts to design and implement public education campaigns regarding the health risks associated with exposure to household air pollution. Promoting improvements to kitchen ventilation and the use of improved cooking stoves, which will help to mitigate the detrimental effects of indoor air pollution on child growth impairment and its long-term effects.

## Background

Stunting in children refers to the impaired linear growth and development, which is frequently, brought on by a persistently poor nutrition, recurrent infections and chronic diseases or poor health conditions [1]. A child is referred to as Stunted when his/her height/length for age Z score is less than –2 SD from the median value of the WHO reference population [2]. In 2022, an estimated 148.1 million under-five children are stunted globally, and more than half of these children reside in developing countries [3]. According to Ethiopia's recent demographic and health survey, 37% of children under five are stunted, and 12% are severely stunted [4].

Stunting imposes several short- and long-term impacts on children and societies [5, 6]. Stunting not only raises the risk of morbidity but also hinders cognitive growth and learning capacity [7–11], lowers productivity [12, 13], raises the risk of infections [14], increases the risk of obstetric complications during adulthood in girls [10, 15], and raises the risk of future non-communicable diseases [8, 16]. Ethiopia's high stunting rate has had a negative impact on the country's socioeconomic development, most notably on health, education, and productivity, and has seriously harmed the country's human capital, on which the economy depends [17]. According to a study on the cost of hunger in Ethiopia, 67% of the adult population suffered stunting as children, making them less productive and affecting their contribution to the national economy, leading to enormous annual economic loss (16.5 percent of the GDP) [18]. Furthermore, stunting is also linked to 24% child mortality, 16% primary school repetition, increased dropout rates, and lower schooling achievement [18].

It is imperative to understand how the nutritional outcomes of children develop through contextualized models, such as using the conceptual model developed by UNICEF [19]. This framework analyzed factors in multiple layers: immediate, underlying, and basic causes of malnutrition. The immediate factors include suboptimal dietary intake, diseases reflecting the underlying social, economic, and nutrition and health service-related conditions. The underlying factors are portrayed as a result of distal or basic determinants such as economic, political, and ideological structures [20]. In line with this model, the literature has documented inadequate nutrition [21–23], illness [1, 24, 25], unclean and unhygienic living conditions [25], lack of access to adequate healthcare for children and their mothers [26], poor childcare and feeding practices [27, 28], poverty [22, 29, 30], and the education status of parents [31, 32] as relevant drivers of stunting in most settings. Apart from these classic drivers, several studies have demonstrated a substantial role of air pollution in childhood stunting [33–40].

Burning solid fuel for cooking is one of the main sources of indoor air pollution (IAP) [41]. Solid fuels, such as charcoal, wood, animal dung, and crop residues, are used as the primary energy source for household cooking, lighting, and heating by approximately two-thirds of the world's population, with the vast majority, approximately 95%, living in developing countries [42–44]. When biomass fuel is burned inefficiently, harmful health-damaging pollutants such as inhalable particulate matter (PM2.5 and PM10), carbon monoxide (CO), carbon dioxide (CO2), a variety of hydrocarbons, and oxygenated and chlorinated organic compounds are released [45, 46]. LMIC nations, particularly those in the Sub-Saharan region, have the highest exposure to HAP due to the widespread use of polluting fuels for essential daily needs in homes with inadequate ventilation and unimproved cooking stoves [47–53]. Family members sharing spaces in homes with domestic animals and overcrowding also increase people's exposure to HAP and its detrimental effects on their health [54].

Children are frequently carried on their mothers' laps or backs while they cook and spend much time in the kitchen, which exposes them to the effects of HAP [55, 56]. Furthermore, they have a higher rate of breathing, absorption, and retention of toxic substances from the air than adults [33, 57, 58]. A recent study conducted in four countries, namely, Ethiopia, India, Peru, and Vietnam, reported that children living in homes where solid fuel is used are more likely to have lower height for age Z scores (HAZ) than children living in homes where clean fuels are used [34]. Similar studies investigating the impact of solid fuel use on childhood stunting reported a strong correlation between solid fuel use and childhood stunting [34, 37, 40, 59, 60]. Furthermore, a systematic review and meta-analysis on air pollution and stunting by Pun V et al. (2019) reported that children exposed to high levels of HAP had up to a 90% increased risk of stunting [59]. Another meta-analysis also verified increased odds of both moderate stunting (OR 1.27, 95% CI 1.12 to 1.43) and severe stunting (OR 1.55, 95% CI 1.04 to 2.30) and exposure to household air pollution [61, 62].

Globally, 93% of all children, including 630 million under five, live in polluted environments and are thus exposed to unsafe levels of air pollution [42]. In Sub-Saharan Africa including Ethiopia, 98% of children are disproportionately affected by air pollution due to widespread uses of biomass fuels for cooking, putting them at a higher risk of disease and death [63, 64]. According to a WHO report, HAP from solid fuel use caused an estimated 3.8 million premature deaths in 2016 and 600, 000 of these deaths occurred among children under the age of five [55]. In Ethiopia, HAP caused 50,320 deaths per year, accounting for nearly 5% of the national disease burden [65].

Ethiopia has a high rate of exposure to HAP due to widespread use of polluting biomass fuel. According to national census information or energy use statistics 97% of the households rely predominantly on solid fuels for their energy needs [50]. In poorly ventilated kitchens that use biomass fuels and unimproved stoves in Ethiopia [66], women and their young children are heavily exposed [67] to smoke for prolonged periods of time while cooking predisposing them its deleterious health impact including impaired linear growth. However, studies on stunting had mostly focused on dietary consumption, childcare, feeding practices, sanitation, and hygiene status as major causes of childhood stunting whereas, a rising body of literature has indicated the potential correlation between air pollution and child linear growth impairments [1, 38, 50, 51].

Despite the high prevalence and significant impact of stunting and the widespread use of solid fuel in Ethiopia, evidence of the association between household air pollution and stunting is overlooked. Hence, the current study is imperative to elucidate the impact of HAP (defined as solid fuel use for cooking) on child linear growth in the current study population.

## Methods

### Study design and setting

A prospective cohort study was conducted among under-five children in Jimma town. Jimma town is located 352 km southwest of the capital city, Addis Ababa, Ethiopia. The town has an estimated population density of 239,430, divided into ten urban and four rural administrative units/kebele [68].

### Study population

The study population consisted of two groups: under five children who lived in households using solid fuel for cooking (exposed group) and those who lived in clean fuel-using households (unexposed group). The exposed groups were selected from three rural kebeles, namely, Kofe, Babala, and Garuke, while the unexposed groups were selected from two urban Kebeles, Awetu Mendera, and Ginjo Guduru. An exposed group was defined as children living in households using solid fuels such as wood, crop residues, charcoal, and animal dung as a primary source of energy for cooking. In contrast, the unexposed group was defined as those children living in households primarily using clean fuel (electricity) as energy sources for cooking.

### Sample size determination and sampling procedure

The sample size was calculated using G-Power V.3.1.9.7 software, assuming an equal number of allocations for the two groups (1:1), 95% confidence level, 90% power, a design effect of 1.5, medium effect size of 0.5, and 10% nonresponse rate. Taking the average HAZ score of 1.5 in exposed groups (solid fuel users) and -1.3 in non-exposed groups (clean fuel users) from a longitudinal data analysis in LMICs [38]. The final sample size was 280 (140 exposed and 140 non-exposed groups).

**Fig. 1:**
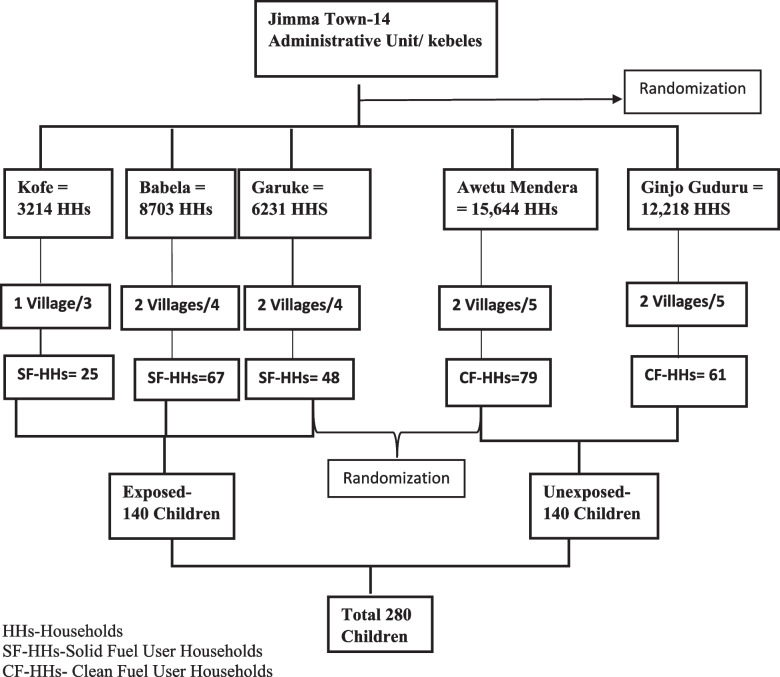
Schematic diagram showing sampling procedure

A multistage sampling technique was used to select the study participants, ensuring randomization at the following levels. In the first stage, one-third of the study kebeles were selected randomly using a lottery method, followed by a selection of villages randomly from each kebele chosen in the second stage. Finally, households were selected from the villages as the third sampling stage using a systematic random sampling technique. The household selection was undertaken using probability proportionate to size criteria. Finally, children under the age of five were selected from each eligible household. Index Child was chosen using a lottery method for families with more than one under-five children (Fig. 1).

### Data collection and measurements

Data were collected through a face-to-face interview using an interviewer-administered pretested structured questionnaire. Trained data collectors (nutritionists, nurses, and environmental health) were involved in data collection. The questionnaire included sociodemographic, socioeconomic, wealth index, housing condition and fuel use pattern, water, hygiene, and sanitation (WASH) condition, immunization and health service utilization, and dietary data. The questionnaire was initially prepared in English and translated into the local language, Afan Oromo. A previously validated Food Frequency Questionnaire (FFQ) containing 28 food items most commonly consumed in the community was used to assess dietary practice [69]. The 28 food items of the food frequency questionnaire were grouped into nine food groups [70, 71]. The FFQ was pretested, and necessary modifications were made before actual data collection.

The Household Food Insecurity Access Scale (HFIAS), developed and validated for Ethiopians, was used to analyze household food security status. The HFIAS consists of two types of related questions. The first part consisted of nine occurrence questions that asked whether a specific condition associated with the experience of food insecurity ever occurred during the previous four weeks (30 days), followed by a frequency-of-occurrence question, which asked how often a reported condition happened during the last four weeks and had a score ranging from 0 to 27 [72]. Furthermore, the Household Food Insecurity Access-related Scale Score, Conditions, Domains, and Prevalence were analyzed. Finally, households ware categorized into food secure and insecure households and then food insecure households were further categorized as mild, moderately, and severely food insecure status. Information were collected by interviewing mother and caretakers of the children.

### Measurement of household air pollutants

The concentrations of particulate matter (PM2.5, PM10) and air pollutants (CO_2,_ CO, and VOC) were measured to assess the exposure level of the households to indoor air pollutants using the Laser PM2.5 Meter-5800D/5800E and Aeroqual's ™ Series 500 portable air quality monitor device [73]. Measurements were performed in the kitchen during cooking when the fire was lit. The monitoring equipment was positioned approximately 1 m above the ground and within 1 m from the cooking stove. The device is adjusted in the real-time measurement and the monitors were calibrated to a zero filter before and after each sampling period.

### Anthropometric measurements

All anthropometric measurements were conducted at baseline and end line after one year. For children up to 24 months of age, crown- heel length was measured with the subject lying supine or face-up. Recumbent length was measured using a stationary headboard and moveable footboard perpendicular to the backboard. The device’s measuring scale had its zero end at the headboard's edge, allowing the child's length to be read from the footboard. Similarly, stature, or standing height, was measured using a stadiometer for subjects two years and older. During measurement, the subjects stood barefooted with heels together, arms to the side, legs straight, shoulders relaxed, and heads in the Frankfort horizontal plane looking straight ahead. Heels, buttocks, scapulae (shoulder blades), and the back of the head were placed against the vertical surface of the measurement board. In measurements of length and stature, a reference was made by positioning the head in the Frankfort horizontal plane and recording it to the nearest 0.1 cm [74, 75]. Children were weighed using an electronic scale to the nearest 0.1 kg. The subjects stood still in the middle of the scale's platform without touching anything and with the body weight equally distributed on both feet and wearing light clothes and shoes off. Anthropometric index for height-for-age, Z score)) were generated using WHO “AnthroPlus” software [76]. To avoid individual variability during anthropometric measurements the same equipment and observer were used for all subjects. Further, to assess the variability of measurements, we calculated the coefficient of variation (CV) below 0.3. All equipment’s for anthropometry and air pollutant measurement were calibrated to insure reliability on each day before measuring each child based on manufacturer instruction [77, 78].

### Data processing and analysis

The collected data were checked, coded, and entered into Epi-Data version 3.1 and then exported to SPSS version 25 software for analysis. Chi-square tests for categorical variables and independent sample t tests for continuous variables were used to determine any statistically significant differences in prevalence and mean between the two groups. Principal component analysis (PCA) was performed to analyze child dietary practices from FFQ data containing 28 food items usually consumed in the community. A Varimax rotation was applied, the Kaiser–Meyer–Olkin measure of sampling adequacy was meritorious (0.86), and Bartlett’s test of sphericity was significant (*p* < 0.05). The variables with communality scores > 50% were retained in the analysis and explained more than 60% of the variance. The factor with the highest eigenvalue was taken and converted into tertiles: low, middle, and high dietary practices. Similarly, the household wealth index was generated from the data using PCA and grouped into wealth tertiles: poor, middle, and rich. Z scores of the anthropometric indices (height-for-age) were computed using WHO AnthroPlus 2007 software [76].

### Difference in differences (DID) test

Difference-in-differences (DID) analysis for different anthropometric measures was used to evaluate the effects of solid fuel on the growth trend of children under the age of five. Accordingly, anthropometric indices of study participants were compared based on exposure status at baseline and end line. First, differences between the end line and baseline measurements were computed for each study group separately by subtracting the baseline score from the end line. Then, the difference in these differences (DID) was analyzed to check whether the growth pattern of the children was different between the exposed and unexposed groups. The difference in differences (DID) estimators for comparison of changes in the outcome (HAZ score) from baseline to end line in exposed (solid fuel (SF)) and non-exposed (clean fuel (CF)) households were computed as:

$$\Delta DID=E[\left(Y1\_SF-Y0\_SF\right)-\left(Y1\_CF-Y0\_CF\right)]$$

where Y_0_ and Y_1_ indicate outcomes at baseline and end line, respectively, and SF and CF represent exposed (SF) and non-exposed (CF).

The difference in difference (DID) linear regression was performed to assess the impact of HAP exposure on the HAZ score.

$$y=\beta 0+\beta 1*Fuel(SF)+\beta 2*post+\beta 3*Fuel*post+\varepsilon$$

**where:**

Y: Is the outcome variable.

Fuel (Treatment): is a dummy variable indicating exposure (= 1) and control (= 0); it represents the difference between fuel1 and fuel0 at baseline.

SF: Solid fuel is exposure (treatment indicator).

Post is a dummy variable indicating baseline (= 0) and end line (= 1) exposure (treatment): it represents how much the average outcome of fuel0 has changed in the end line period.

Fuel*Post is the difference in difference estimator. It represents how much the average outcome of fuel1 has changed in the period compared to what would have happened to the control.

ℇ is the error term.

The assumptions of the model (normality and homoscedasticity of error terms and linearity of relationship) were assessed using partial plots and found to be satisfactory. In all multivariable models, the absence of multicollinearity was evaluated using the variance inflation factor and found to be within the acceptable range (variance inflation factor < 10). Significant differences were declared at P < 0.05.

## Results

### Baseline demographic and socioeconomic characteristics

This study involved 280 under-five children (140 from solid fuel user households = exposed and 140 from clean fuel users = unexposed). The mean (SD) age of exposed and unexposed children was 3.0 (1.3) and 3.1 (1.2) years, respectively. Nearly 71.4% of mothers in solid fuel user households and 26.4% in clean fuel user households did not attend a formal education (Table 1).

**Table 1:** Demographic and socioeconomic characteristics of the study households at baseline, Jimma Ethiopia, 2023

Variables	Clean Fuel	Solid Fuel	*P* value
Mean (SD) age	3.1 (1.2)	3.0 (1.3)	0.230
Mean (SD) family size	4.3 (1.8)	5.2 (1.8)	< 0.001*
**Sex**			
Male	78 (55.7)	67 (47.9)	0.232
Female	62 (44.3)	73 (52.1)	
**Wealth index**			
Low	40 (28.6)	52 (37.1)	0.044*
Medium	39 (27.9)	47 (33.6)	
High	61 (43.6)	41 (29.3)	
**Household head**			
Male	116 (82.9)	122 (87.1)	0.403
Female	24 (17.1)	18 (12.9)	
**Father education**			
No formal education	17 (12.1)	42 (30.0)	< 0.001*
Primary	31 (22.1)	65 (46.4)	
Secondary	53 (37.9)	27 (19.3)	
Higher	39 (27.9)	6 (4.3)	
**Mother education**			
No	37 (26.4)	100 (71.4)	< 0.001*
Primary	58 (41.4)	38 (27.1)	
Secondary	36 (25.7)	2 (1.4)	
Higher	9 (6.4)	0 (0.0)	
**Occupation**			
Housewife	92 (65.7)	35 (25.0)	< 0.001*
Farmer	8 (5.7)	105 (75.0)	
Employed	40 (28.6)	0 (0.0)	
**Immunization**			
Fully immunized	101(72.1)	85 60.7	0.105
Partially immunized	34 (24.3)	45 32.1	
Not immunized	5 (3.6)	10 (7.1)	

Values are n (%) unless otherwise specified, *P* values denote *p* < 0.05* (χ2 test)

### Dietary practice and household food insecurity access scale (HFIAS)

In the study, household dietary practice and food insecurity access scale was evaluated. Accordingly, Approximately 66.8% (95% CI 27.5%, 38.9%) of the study participants had poor dietary practices. In both groups, dark green leafy vegetables (DGLV) and egg were consumed more frequently. However, flesh food and egg consumption were higher in the clean fuel group than in their counterparts. There was a significant difference in the intake of starchy and staple foods (*p* = 0.023), legumes, nuts and seeds (*p* < 0.001), dairy (*p* < 0.001), flesh foods (*p* < 0.001), eggs (*p* < 0.001) and other fruits and vegetables (*p* < 0.001) between the exposed and unexposed groups (Table 2).

**Table 2:** Food consumption patterns among children of solid and clean fuel user households in Jimma Ethiopia, 2023

**Food groups**	**Solid Fuel(*n* = 140)**	**clean fuel (*n* = 140)**	
	**n (%)**	**n (%)**	***p***
Starchy and staple foods	78(55.71)	58(41.42)	0.023*
DGLV	133(95)	138(98.57)	0.173
legumes, Nuts, and seeds	127(90.71)	66 (47.14)	< 0.001*
Dairy	43(30.71)	103 (73.57)	< 0.001*
Flesh foods	45(32.14)	133(95)	< 0.001*
Eggs	125(89.28)	140 9140)	< 0.001*
Vitamin A-rich Fruit	40(28.57)	43(30.71)	0.084
Other vitamin A-rich fruits and vegetables	63(45)	62(44.28)	0.794
Other fruits and vegetables	61(45.57)	69(49.28)	< 0.001*

*DGLV* Dark green leaf, and vegetables. *P* values are denoted as *p* < 0.05* (χ2 test)

The total prevalence of food insecurity in the study area was 34.6%. Comparing the two groups, food insecurity was significantly higher in exposed groups than in their counterparts, with a prevalence of 52.1% (95% CI: %) (*p* < 0.001). Concerning the degree of household food insecurity, 31%, 33%, and 6.4% of the exposed group were severely, moderately, and mildly food insecure, respectively (Table 3). Concerning the household's food insecurity access scale, approximately 52.14% of respondents in exposed households experience anxiety and uncertainty about food supply. Similarly, 51.43% of households encountered insufficient food quality, and 45% had inadequate food intake. Likewise, 14.3%, 17.1%, and 14.3% of respondents in exposed households experience anxiety and uncertainty, insufficient food quality, and insufficient food intake, respectively.

**Table 3:** Household Food Insecurity Access Scale (HFIAS) of Study participants in Jimma Ethiopia, 2023

Variables	Clean Fuel	Solid Fuel	*P* value
**Dietary practice**			
Low	6 (4.3)	87 (62.1)	< .001
Medium	45 (32.1)	49 (35.0)	
High	89 (63.6)	4 (2.9)	
**HFIAS score**			
Mean (SD)			
** HFIAS prevalence**			
Food Secure	116 (82.9)	67 (47.9)	< .001
Mildly Food Insecure Access	4 (2.9)	9 (6.4)	
Moderately Food Insecure Access	4 (2.9)	33 (23.6)	
Severely Food Insecure Access	16 (11.4)	31 (22.1)	
**HFIAS Domains**			
Anxiety and uncertainty	20 (14.3)	73(52.14)	< .001
Insufficient food quality	24(17.1)	72(51.43)	
Insufficient food intake	20(14.3)	63 (45)	

Values are n (%) unless otherwise specified, *P* values denote *p* < 0.05* (χ2 test)

### Household water, hygiene and sanitation (WASH) conditions

The Household hygiene and sanitation conditions (WASH) practices wash conditions of the households are given in Table 4 Approximately 46.4% of exposed households had access to improved drinking water sources, 37.1% had access to improved sanitation, and 22.1% had good hygiene practices. Similarly, all respondents in unexposed households had access to improved drinking water sources, whereas 60% had improved sanitation, and 44.3% had good hygiene practices.

**Table 4:** Household drinking water sources, toilet facilities, and sanitation practices of study participants in Jimma Ethiopia, 2023

Variables	Clean Fuel	Solid Fuel	*P* value
**Drinking Water (%)**			
Tap water/piped into Dwelling	140 (100.0)	2 (1.4)	
Dug Well	0 (0.0)	23 (16.4)	
Spring Water	0 (0.0)	52 (37.1)	
Public Tap/Stand Pipe	0 (0.0)	41 (29.3)	
Borehole	0 (0.0)	22 (15.7)	
**Drinking Water sources (%)**			
Unimproved Sources	0 (0.0)	75 (53.6)	< .001
Improved Sources	140 (100.0)	65 (46.4)	
**Toilet facilities (%)**			
No latrine/Bush	0 (0.0)	32 (22.9)	
Shared Public Facility	22 (15.7)	11 (7.9)	
Pit latrine with slab	60 (42.9)	40 (28.6)	
Composting toilet	13 (9.3)	14 (10.0)	
Flush/pour latrine	11 (7.9)	0 (0.0)	
Open Pit latrine	34 (24.3)	43 (30.7)	
**Sanitation Status (%)**			
Improved	84 (60.0)	52 (37.1)	< .001
Un Improved	56 (40.0)	88 (62.9)	
**Hand washing practice after toilet (%)**			
No	36 (25.7)	71 (50.7)	
Yes Sometimes	104 (74.3)	69 (49.3)	
Yes Usually			
Hand washing practice after toilet using (%)			
None	36 25.7	71 (50.7)	< .001
Soup and or Detergent	62 44.3	31 (22.1)	
Water only	42 30.0	38 (27.1)	
**Hygiene Practice (%)**			
Good Hygiene Practice	62 (44.3)	31 (22.1)	0.000
Poor Hygiene Practice	78 (55.7)	109 (77.9)	

Values are n (%) unless otherwise specified, *P* values denote *p* < 0.05* (χ2 test)

### Household concentration of indoor air pollutants

The household exposure variables were also evaluated in the study and the median concentrations of pollutants were as follows: PM_2.5_, 293.95 µg/m^3^ (IQR: 770.26); PM_10,_ 270.85 µg/m3 (IQR: 1893.38), CO_2_, 577.50 mg/m3 (IQR: 350), CO, 7.90 mg/m3 (IQR: 8.20), and VOC, 1O77.50 mg/m3 (IQR: 861). A statistically significant difference was observed in the concentration of indoor air pollutants between solid and clean fuel user households (*p* < 0.001) (Table 5).

**Table 5:** Household concentration of indoor air pollutants and exposure indicators in Jimma Ethiopia, 2023

Variables		Clean Fuel	Solid Fuel	*P* value
**Indoor air pollutants**				
PM2.5 µg/m^3^	Median(IQR)	99.00 (75.80)	905.10(336.50)	< .001
	Mean rank	70.88	210.12	
PM10 µg/m^3^	Median(IQR)	119.70(73.10)	1999(1827.30)	< .001
	Mean rank	70.95	210.50	
CO2 mg/m^3^	Median(IQR)	507.00(123)	893.00(1186)	< .001
	Mean rank	95.64	185.36	
CO mg/m^3^	Median(IQR)	7.00(4.60)	11.25(20.75)	< .001
	Mean rank	81.67	118.52	
VOC mg/m^3^	Median(IQR)	817(347)	1550.50(583)	< .001
	Mean rank	85.33	195.67	

*PM2.5* Particulate matter < 2.5 µm in diameter. *PM10* Particulate matter < 10 µm in diameter. *CO2* Carbon dioxide. *CO* Carbon monoxide. *VOC* Volatile Organic Compound. *IQR* Interquartile Range

^*^P values refer to the difference between the two fuel types compared. Tested with the Mann‒Whitney U test for medians

### Relationship between household fuel types and children linear growth

The difference in differences (DID) estimator was used to examine the Effect of fuel type used on the linear growth of under- five children. In difference in differences (DID), the HAZ score of study participants was compared based on exposure status at baseline and end line (Table 6). The results showed a significant difference in the differences in the mean HAZ score between the two groups. The mean values of baseline and end line differences of HAZ score between the exposed and unexposed children were 0.54 (*p* < 0.005). Unexposed children had a significantly high difference between the end line and baseline mean HAZ score (Table 6).

**Table 6:** Differences in differences between baseline and end-line measurements of anthropometric indices among clean and solid fuel types for children in Jimma, Ethiopia, 2023

	**Clean Fuel**			**Solid Fuel**			**DID (Solid-clean)Mean(SE)**	***P* value**
	Baseline Mean(SE)	End line Mean(SE)	Difference (EL-BL) Mean(SE)	Baseline Mean(SE)	End line Mean(SE)	Difference (EL-BL) Mean(SE)		
**HAZ**	-0.40(1.21)	0.09(1.14)	0.48(0.86)	-1.41(1.42)	-1.47(1.30)	-0.06(0.84)	-0.54(0.21)	0.011**

^**^Significant at 0.05 level of significance, *EL* End-line mean, *BL* Baseline mean, *DID* Difference in difference (mean difference of SF-mean difference of CF), *SE* Standard error

To account for potential confounders, we computed a linear regression analysis. In an unadjusted model (Model 1), compared with the clean fuel type, the mean difference in the difference in the HAZ score of the solid fuel type was lower by 0.54 (-0.543, 95% CI -0.97, -0.12). The beta coefficient remained negative after adjusting for age and sex (Model 2 -0.543, 95% CI -1.373, -0.563) and sociodemographic variables (Model 3: -0.543, 95% CI -1.362, -0.575). In the final model, adjusted as model four and, wealth quantile, dietary practice, WASH, and household food insecurity access scale, there was a significant difference in favor of clean fuel type on HAZ-score indices (Model 4: -0.543, 95% CI -1.357, -0.579) (Table 7).

**Table 7:** Multivariable linear regression models predicting mean baseline to end-line differences in the differences in HAZ score among the clean and solid fuel types for the children in Jimma, Ethiopia, 2023

Models	${\varvec{\beta}}$(95% CI) in Z score	Covariates
Model 1	-0.543 (-0.97, -0.12)*** SE = 0.211	Unadjusted
Model 2	-0.543 (-1.373, -0.563)*** SE = 0.206	Age of child and sex
Model 3	-0.543(-1.362, -0.575)*** SE = 0.200	Age of child, family size, sex, wealth index, father’s education, mother’s education, and occupation
Model 4	-0.543 (-1.357, -0.579)*** SE = 0.198	Age of child, family size, sex, wealth index, father's education, mother's education, occupation, Dietary practice, HFIAS prevalence, drinking water sources, sanitation status, and hygiene practice

^*^Significant at *P* < 0.05

^**^Significant at *P* < 0.01

^***^Significant at *P* < 0.001, all $\beta$ coefficients (95% CI) were from multiple linear regression analysis and related to the non-exposed groups. Model 1, Unadjusted; Model 2, age and sex; Model 3, sociodemographic factors; Model 4, Biological & sociodemographic factors, HFIAS, Dietary, WASH (fully adjusted)

The linear regression analysis of this study found that the sex of the child, the father's education, and hygiene practices were relevant factors for the HAZ score at the 0.05 significance level. Higher HAZ scores were observed among female child (β: = 0.48, 95%CI: 0.28, 0.69), Child with father attended higher education (β: = 0.304 95%CI: 0.304, 95% CI 0.19, 0.41) as compared to male gender and those who did not attend a formal education, respectively. In contrast, child living in households with poor hygiene practices had lower HAZ score (β: -0.226, 95% CI: -0.449, -0.003).

## Discussion

This study examined the nutritional impact of exposure to HAP on the linear growth of children under five years of age. The results indicated that exposure to HAP was inversely related to linear growth. Compared with children living in households that use clean fuel for cooking, a lower average HAZ score (-0.54) among children in solid fuel-using households (*p* < 0.001) was observed. Furthermore, in the multivariable linear regression fully adjusted model for all possible covariates, the coefficient on the HAZ score was negative and statistically significant (coefficient: -0.54, *p* < 0.001).

These findings were corroborated by the results of many studies from similar settings, such as India [34, 38, 60], Nepal [39], Peru, Vietnam, and Bangladesh [37, 39, 40]. These studies found that children living in households where solid fuel is used are likely to have lower height for age Z scores (HAZ) than those living in households where clean fuel is used for cooking. They also reported a significantly higher prevalence of stunting among children living in households using solid fuels than among those using clean fuels. In line with our findings, the available systematic reviews and meta-analyses confirmed a strong correlation between household air pollution exposure and stunting [61, 62]. Additionally, a systematic review and meta-analysis on the relationship between air pollution and stunting found that children who were exposed to household air pollution had a higher risk of stunting, ranging from 13 to 19% [59, 79].

HAP exposure impairs a child's growth and development through its direct and indirect effects on various biological systems [80–82]. Due to their small size, air pollution particles are inhaled into the lungs, infiltrate the bloodstream, and reach various body organs, resulting in increased oxidative stress, systemic inflammation, and altered immune function [83–86]. Prenatal exposure to air pollution can induce reactive oxygen species (ROS), which can cause cell damage, including DNA, protein, and lipids [87, 88]. Reactive oxygen species (ROS) regulate several signaling pathways through interactions with critical signaling molecules, affecting various cellular processes, such as proliferation, metabolism, differentiation, and survival [89]. More importantly, high levels of ROS cause mitochondrial dysfunction, reduced telomere length, inflammation, and potentially poor fetal growth [90]. Reactive oxygen species also result in epigenetic modifications and reduced DNA methylation [38, 39]. Modulation in DNA methylation is assumed to be one of the epigenetic mechanisms by which air pollution leads to poor fetal growth [91–93].

Likewise, exposure to air pollutants is associated with increased levels of proinflammatory mediators in the systemic circulation [94] (11), which affects bone metabolism through a specific effect of cytokines such as TNFα, IL-1β, IL-6, and IL-17 on osteoblast and osteoclast differentiation and function, leading to growth suppression [95–97]. Additionally, there are direct relationships between systemic inflammation, growth hormone (GH) signaling, and linear growth [98]. Higher systemic inflammation is related to GH resistance [99], higher systemic levels of growth hormone [100], lower hepatic production of insulin-like growth factor (IGF-1) and IGF binding-protein-3 (IGFBP-3), lower systemic levels of IGF-1 and IGFBP-3 [101], and poor responsiveness of the growth plate that results in slower and impaired growth [102–105]. Furthermore, air pollution might impair linear growth through recurrent incidents of febrile respiratory infection [83], which affects immune activity and leads to increased metabolic requirements, reduced dietary intake, increased catabolism, altered metabolism and nutrient imbalance, and hence impaired growth [83, 106]. Air pollution can also cause vitamin D deficiency, which is essential for regulating bone metabolism, immune function, and growth [107–109].

Household air pollution is a major health concern for children's growth and development in low- and middle-income countries (LIMICs) [45, 64]. The predominant use of solid fuel for basic energy needs in poorly ventilated kitchens and unimproved traditional cooking appliances, as in the majority of households in LMICs in the setting, increases the likelihood of exposure to harmful full air pollutants [66]. Furthermore, exposures experienced by household members, particularly women and young children who are frequently carried on their backs and spend a significant portion of their time indoors, have been measured to be many times higher than World Health Organization (WHO) guidelines [50], predisposing them for the negative health impacts of HAP including impaired childhood linear growth and developments.

Given the large size of the stunted population, all possible factors contributing to child stunting should receive relevant policy attention. Previous studies conducted in most low-income countries have focused on dietary consumption, childcare practices, water, sanitation, and hygiene (WASH) as major drivers of childhood stunting [19]. In contrast, air pollution has been largely overlooked in the LIMICS setting. This finding suggests that future nutrition policymakers should consider the impact of indoor air pollution on child linear growth and implement targeted interventions on programs that help reduce exposure to health-damaging indoor air pollutants, such as efforts to improve household air quality, reduced use of polluting solid fuels, promotion of cleaner energy, and access to improved cooking stoves. More importantly, it provides insight into a broader approach that addresses the causes and potential interventions to significantly reduce the burden of childhood stunting, which also aids the country's efforts to meet SDGs-related targets, particularly goals two (zero hunger) and sixty (climate action). Furthermore, it serves as a foundation for future research on the relationship between solid fuel use and stunting in children under the age of five, such as determining the causal role of solid fuel use on childhood stunting with randomized controlled trials.

### Limitation of the study

Changes in the dietary behavior or sociodemographic characteristics in the end line was not accounted in the presented study. However, we believe measures of FFQ and wealth index may not necessary change in a population over short period of time.

## Conclusion

Exposure to indoor air pollution was inversely related to linear growth. Furthermore, sex, educational status and hygiene were found relevant predictors of linear growth. In such a setting, appropriate policy action needs to be kept in place to mitigate the negative impact of indoor air pollution on child growth impairment and its long-lasting consequences. Additionally, there is a need to step up efforts to design and implement public education campaigns regarding the health risks associated with exposure to household air pollution. Promoting improvements to kitchen ventilation and the use of improved cooking stoves, which will help to mitigate the detrimental effects of indoor air pollution on child growth impairment and its long-term effects.

## Data Availability

The data presented in this study are available in the article.
